# Similarities and Differences Between Therapy-Related and Elderly Acute Myeloid Leukemia

**DOI:** 10.4084/MJHID.2011.052

**Published:** 2011-11-28

**Authors:** Francesco D’Alò, Luana Fianchi, Emiliano Fabiani, Marianna Criscuolo, Mariangela Greco, Francesco Guidi, Livio Pagano, Giuseppe Leone, Maria Teresa Voso

**Affiliations:** Istituto di Ematologia, Università Cattolica Sacro Cuore, Rome, Italy

## Abstract

Acute myeloid leukemia (AML) is a clonal disorder of the hematopoietic stem cell, typical of the elderly, with a median age of over 60 years at diagnosis. In AML, older age is one of the strongest independent adverse prognostic factor, associated with decreased complete response rate, worse disease-free and overall survival, with highest rates of treatment related mortality, resistant disease and relapse, compared to younger patients. Outcomes are compromised in older patients not only by increased comorbidities and susceptibility to toxicity from therapy, but it is now recognized that elderly AML has peculiar biologic characteristics with a negative impact on treatment response.

In older individuals prolonged exposure to environmental carcinogens may be the basis for similarities to therapy-related myeloid malignancies (t-MN), which result from toxic effects of previous cytotoxic treatments on hematopoietic stem cells. Age is itself a risk factor for t-MN, which are more frequent in elderly patients, where also a shorter latency between treatment of primary tumor and t-MN has been reported. t-MN following chemotherapy with alkylating agents and elderly AML frequently present MDS-related cytogenetic abnormalities, including complex or monosomal karyotype, and a myelodysplastic phase preceding the diagnosis of overt leukemia. Similarly, t-MN and elderly-AML share common molecular abnormalities, such as reduced frequency of NPM1, FLT3 and CEBPA mutations and increased MDR1 expression.

Given the unfavorable prognosis of elderly and t-MN and the similar clinical and molecular aspects, this is a promising field for implementation of new treatment protocols including alternative biological drugs.

## Introduction

Acute myeloid leukemia (AML) is year and represents 90% of acute leukemia in adults, mainly a disease of older adults. Worldwide, AML while it is rare in children. The AML incidence rises affects approximately 3 to 4 individuals in 100,000 per rapidly after 50 years, with a median age at diagnosis of 67 years.^1^

Therapy-related AML (t-AML) accounts for 10 to 20% of AML cases in adults. The incidence of t-AML is increasing due to longer life expectancy of cancer survivors. Therapy-related myeloid neoplasms (t-MN), including t-AML and therapy-related myelodisplastic syndromes, are now recognized as distinct nosographic entities according to the 2008 WHO classification of tumors of Hematopoietic and Lymphoid Tissues (2008).^2^,^3^

Age is a risk factor also for t-AML, which occurs at a higher median age compared to de novo AML.^4^,^5^ Interestingly, patients with breast cancer treated with adjuvant chemo/radiotherapy who develop t-MN are older at breast cancer diagnosis (mean age 60.2 years versus 54.5 years; *P* = 0.01),^6^ and older age at the primary cancer diagnosis has been associated to shorter latency to t-MN development.^7^ History of multiple cancer and familial history of neoplastic diseases represent additional risk factors for t-MN.^6^

This review will focus on similarities between elderly and therapy-related AML, both from the clinical and biological point of view. These diseases are commonly associated to worse prognosis related both to tumor biology and host-related factors. Tumor biology is characterized by adverse prognostic signature affecting cytogenetics, molecular genetics, epigenetics, GEP, and multidrug resistance (Table 1). Similarly, compared to *de novo* AML in younger patients, elderly patients and those with t-MN more often present significant comorbidities, reduced tolerance to treatment, higher incidence of treatment-related complications and are commonly excluded from treatment protocols.

In this review, we will use the terms “AML in older patients” and “elderly AML” to indicate AML occurring in individual aged 60 years or more. Changes of leukemia features will be described according to patients’ age considering different age intervals. Nevertheless, aging is a continuum of changes affecting not only individuals, but also several aspects of diseases. In particular, leukemia biological and clinical features change gradually with aging without any sharp distinction or any universally accepted temporal watershed to define distinct disease subsets.

## Morphological and Cytogenetic Aspects

Morphological/laboratory characteristics as well as clinical course present several similarities between t-AML and elderly AML. Cytological and cytochemical differences have been described between “young” and elderly AML, indicating a less differentiated phenotype for “elderly” blasts.^8^ Elderly AML usually presents with lower white blood cell (WBC) counts compared to younger patients (12,510/μl versus 19,800/μl, *P* < .001).^9^ Similarly, t-AML have lower WBC count at diagnosis, compared to *de novo* AML (7400/μl versus 12500/μl, respectively, *P* = 0.003).^4^

The WHO Classification of Myeloid Malignancies identifies AML with myelodysplasia-related changes (AML-MRC) as a distinct AML subclass when at least one of the following prerequisites is fulfilled: a history of secondary AML arising from a previous MDS or MDS/MPN, AML with MDS-related cytogenetic abnormalities, and/or AML with multilineage dysplasia (MLD).^3^ The presence of MDS-related cytogenetic abnormalities and/or a MDS or MDS/MPN history is associated to older age (69.8 years versus 65.6 years, *P* = 0.011), lower median WBC count (6300/ μl versus 13800/μl, *P* < 0.001) and worse median EFS (10.7 months versus 16.9 months; *P* = 0.005) and OS (16.8 months versus not reached; *P* = 0.001).^10^ This confirms the impact of a previous history of MDS or of typical cytogenetics, as one of the main biological factors in AML.

Two distinct t-AML subtypes have been described according to the previous cytotoxic therapy.^2^,^3^ Those t-AML occurring 5–10 years after the exposure to alkylating agents and/or ionizing radiation present several similarities with elderly AML. These patients commonly present a myelodysplastic phase (t-MDS) and bone marrow failure with one or multiple cytopenias before evolving into overt leukemia. This subset of t-AML is commonly associated with MDS-related cytogenetic abnormalities, such as monosomies or partial deletions of chromosome 5 and/or 7. On the contrary, a smaller subset of t-AML occurring at 1–5 years after exposure to topoisomerase II inhibitors, does not have a myelodysplastic phase but presents as overt leukemia and is often associated with balanced chromosomal translocations. It could be hypothesized that limited amounts of genetic and epigenetic events could be implicated in the pathogenesis of the latter type of t-AML, while t-MN following alkylating agents requires a longer latency period and probably a multistep accumulation of genetic and epigenetic events that finally result in overt leukemia.^2^,^3^

Cytogenetics has been indicated as the most powerful outcome predictor in AML, independent of age and other potential confounders.^11^ Elderly AML and t-AML share common cytogenetic aspects. Elderly AML is associated to increasing incidence of unfavorable complex karyotypes and a decreasing incidence of favorable balanced chromosomal translocations.^9^,^12^,^13^ In a large study evaluating the age-specific incidence of cytogenetic abnormalities in 2555 patients with AML between 21 and 70 years of age, adverse cytogenetic profiles were disproportionately seen in elderly patients, while balanced translocations decreased and unbalanced aberrations increased with age.^12^ Similarly, in 1284 patients with AML aged 18 to 85 years, a lower incidence of favorable karyotypes and a higher incidence of unfavorable complex karyotypes were seen in patients aged over 60 years.^9^

Monosomal kayotype (MK), defined as 2 or more distinct monosomies or a single monosomy in the presence of other structural abnormalities, identifies a distinct subset of AML with an extremely poor prognosis and a 4-year OS of 3–4%, even worse than that of unfavorable cytogenetics.^14^,^15^ The proportion of patients with MK-AML including -5/5q- and -7/7q-increases with age, from 4% in patients younger than 30 years to 20% in those older 60 years of age or more.^15^

In the same line, t-AML frequently present abnormal karyotype (75% versus 51%, *P* < .0001) and adverse cytogenetics (39% versus 19%, *P* < .0001). The frequency of favorable-risk abnormalities is similar in t-AML and *de novo* AML, while the intermediate risk category is less frequent in t-AML. Common poor risk cytogenetic abnormalities significantly enriched in t-AML group include t(9;11), -5/5q-, -7/7q-, abnormalities (17p), complex karyotype and monosomal karyotype.^4^ A trend toward a higher representation of trisomy 8 outside a complex karyotype has also been reported in t-AML.^4^

## Recurrent Molecular Abnormalities

Heterogeneous data have been published about the prevalence of NMP1 mutation in elderly AML. On the other hand, FLT3 internal tandem duplications (ITD) were shown to be less common in elderly compared to younger AML patients (23% and 37%).^16^ Both NPM1 mutation and FLT3 ITD have been reported to be under-represented in elderly normal karyotype AML compared to younger patients (52.1% and 66.4% for NPM1 mutation; 26.6% and 37.2% for FLT3-ITD, respectively).^9^ The frequency of both NPM1 mutations and FLT3-ITD are significantly lower in t-AML compared to *de novo* AML, indicating that secondary leukemogenesis might follow mechanisms different from those seen in *de novo* AML. Nevertheless, in cytogenetically normal AML, no difference was found in the incidence and distribution of mutated NPM1 and FLT3-ITD in t-AML and de novo AML.^4^

TET2 mutations occur in 23% of AML patients and are associated with older age (*P* < .001). The prevalence of TET2 mutations gradually increased with age, from 7% in adults younger than 30 to 32% in patients aged 70 years or older (*P* < .001).^21^ Mutation of at least one copy of the TET2 gene was detected in 49/247 (19.8%) patients with secondary acute myeloid leukemia, including both AML with MRC (n=201) or therapy-related (n=46) leukemias. In this study, TET2 mutations were significantly less frequent in therapy-related (8.7%) than MRC (22.3%; *P* = 0.035) AML.^18^

DNMT3A mutations occur in 22% of AML patients, are highly enriched in the group of patients with an intermediate-risk cytogenetic profile, and are associated to shorter overall survival. Patients carrying DNMT3A mutations have a higher median age at the diagnosis compared to unmutated patients.^19^ There are no data on the incidence of DNMT3A mutation in t-AML.

Isocitrate dehydrogenases IDH1 and IDH2 mutations, which lead to accumulation of 2-hydroxyglutarate in neoplastic cells, are recurrent in AML patients (about 16% of AML patients), particularly in those with normal cytogenetics and NPM1 mutated, and have an unfavorable impact on outcome. Patients carrying IDH mutations present a higher median age at diagnosis compared to IDH wild-type patients.^20^,^21^

CEBPA mutations occur in 6–15% of AML patients and are more common in patients with normal kayotype. Double CEBPA-mutant have a better prognosis compared to wild-type and single mutant. Frequency of double CEBPA mutation decrease by increasing age.^22^ CEBPA mutations are uncommon in t-MN.^23^ RUNX1 mutations occur in 5.6% of AML patients with no difference in frequency among different age groups.^24^ RUNX1 mutation are common in t-MN (15.7% in a cohort of 140 patients) and are commonly associated to previous therapy with alkylating agents and chromosome 7 deletion.^25^ NRAS mutations occur in 10.3% of AML patients with no differences according to age and between de novo and therapy-related AML.^26^ WT1 mutations occur in 6.8% of AML patients and are closely associated with younger age^27^ and are uncommon in t-MDS/AML.^28^

## Disease Behaviour and Biology

Several adjunctive molecular features make elderly and therapy-related AML biologically different from *de novo* AML in younger patients, involving multidrug resistance, telomere shortening, gene expression profiling and gene promoter methylation.

P-glycoprotein (Pgp) is a membrane transporter encoded by the multidrug resistance (ABCB1, MDR1) gene, which traps hydrophobic drugs, including key chemotherapeutic drugs in the plasma membrane of cells and effluxes them using an ATP-dependent process. High Pgp expression is detected in about 50% of AML blast samples, is more common in older (71%), than in younger cohorts (35%) and is associated with poor response to chemotherapy.^29^,^30^ Similarly to elderly AML, secondary leukemias present higher Pgp expression compared to *de novo* AML (61 versus 37, *P* < 0.001),^31^ which may lead to reduced responsiveness to chemotherapy and chemoresistance.

Telomeres are specialized structures composed of TTAGGG repeats and associated proteins located at the end of eukaryotic chromosomes. Telomere repeats are lost with each cell division, eventually leading to genetic instability and cellular senescence when telomeres become critically short. Age-adjusted telomere length in AML patients is significantly shorter than in matched healthy controls. Surprisingly, patients younger than 60 years had significantly shorter age-adjusted telomere length than patients older than 60 years (median: −3.4 vs. −1.7 telomere fluorescence units, TFU; *P*<0.001), while age-adjusted telomere length was found to be significantly shorter in patients displaying an aberrant karyotype than in patients with a normal karyotype (median −3.0 vs. −2.3 TFU; p=0.03).^32^ The development of t-MDS/AML has been associated with and preceded by markedly altered telomere dynamics in hematopoietic cells. Accelerated telomere shortening in patients developing t-MDS/AML is associated to genomic instability and may contribute to leukemic transformation.^33^ In experimental models, irradiation and chemotherapy were able to induce persistent and progressive telomere shortening and DNA damage accrual, reproducing the effects of aging in the hematopoietic system.^34^,^35^

Elderly AML also display a distinct gene expression profiling (GEP) compared to younger AML patients. In a cohort of 525 adult AML, the transcriptome of the 175 oldest patients (median age 59.4 years) compared to that of the 175 youngest patients (median age 31.1 years) revealed 969 differentially expressed probe sets, of whom 477 were up-regulated and 492 down-regulated with increasing age. The Gene Ontology analysis of 145 “Up-with-Age” as well as 257 “Down-with-Age” probe sets revealed that genes involved in biologic processes, as regulation of I-κB kinase NF-κB cascade and immune response were up-regulated with increasing age. On the other hand, biologic processes as establishment or maintenance of chromatin architecture, regulation of cyclin-dependent protein kinase activity, and cell matrix and cell-substrate adhesion were found to be downregulated with increasing age. In particular, among differentially-expressed genes, p16INK4A, encoded by the tumor-suppressor gene CDKN2A, is down-regulated in elderly AML samples, while it is normally induced during the physiologic aging of hematopoietic stem cells.^36^ Similarly, the analysis of oncogenic signaling pathway showed that older patients have a lower probability of E2F and PI3-kinase pathway activation, but a higher probability of RAS, TNF, Src, and EPI pathway activation. Using genomic-derived signatures of anthracycline-sensitivity, elderly patients presented a uniformly anthracycline resistant signature irrespective of tumor biology, while younger patients were much more likely to be sensitive to adriamycin.^37^

According to GEP, two distinct t-AML subgroups have been identified: the first group associated with del5q/-5 shows a higher expression of genes involved in cell cycle control (CCNA2, CCNE2, CDC2), checkpoints (BUB1), or growth (MYC), and loss of expression of the gene encoding IFN consensus sequence-binding protein (ICSBP). The second t-AML group is characterized by down-regulation of transcription factors involved in early hematopoiesis (TAL1, GATA1, and EKLF) and overexpression of proteins involved in signaling pathways in myeloid cells (FLT3) and cell survival (BCL2). Moreover CD34+ cells from patients with t-AML present a more immature profile than normal CD34+ cells.^38^

Epigenetic changes, in particular DNA methylation, are recognized as significant contributors to leukemogenesis, and in particular in therapy-related diseases. By a single gene analysis of promoter hypermetylation of several tumor-suppressor genes, BRCA1 and DAPK1 were found more frequently hypermethylated in therapy-related than in *de novo* AML, while no significant differences were found according to patients’ age.^39^–^42^ In 356 patients studied for concurrent methylation of several promoters, t-MDS/AML were significantly more frequently hypermethylated in 2 or more promoter regions than *de novo* MDS or AML (17% vs 11.5% and 9.5%, p=0.035).^42^ Moreover, the methylation profile of 14,000 promoters in MDS and secondary AML have showed that these diseases display more extensive aberrant DNA methylation involving thousands of genes than normal CD34+ bone marrow cells or *de novo* AML blasts. This aberrant methylation affected particular chromosomal regions, occurred more frequently in Alu-poor genes, and included prominent involvement of genes involved in the WNT and MAPK signaling pathways.^43^

## Treatment Outcome

The most significant similarity between elderly and t-AML is the poor prognosis, compared to younger and *de novo* AML.

Older age is a major adverse prognostic factor for AML in general. Surveillance, Epidemiology, and End Results (SEER) statistics from 1996 to 2002 show 5-year relative survival rates of 34.4% for adults aged under 65 and 4.3% for those over 65 years.^13^ A retrospective analysis of 968 patients with previously untreated AML in 5 Southwest Oncology Group trials compared treatment outcome by stratifying patients into 4 different age groups (<56, 56–65, 66–75, and >75 years). The CR rates decreased from 64%, to 46%, 39%, and 33%, respectively, while the median overall survival was 18.8, 9.0, 6.9, and 3.5 months, respectively. In responding patients, median disease-free survival was 21.6, 7.4, 8.3, and 8.9 months, respectively.^44^ In the same analysis, age had a modest effect in patients with an excellent performance status (PS), but for patients with a PS of 2 or 3, age had a dramatic effect, with 82% of patients older than 75 and a PS of 3 dying within 30 days from induction start.^44^

In the last three decades significant improvement in survival has been mainly observed in younger AML patients. In a large Swedish population-based cohort study including 9729 AML patients diagnosed between 1973 and 2005, one-year survival increased across all age categories, but the improvement in 5- and 10-year AML survival was confined to patients younger than 80 years and was most prominent in the young (<60 years) patient population.^45^

Similar to elderly AML, patients with t-AML are often precluded from intensive treatment due to frequent comorbidities and cumulative toxicities, related to previous cytotoxic treatments. Also when appropriately treated as *de novo* AML, t-AML still has worse outcome. According to the analysis of 6 prospective multicenter treatment trials of the German-Austrian AMLStudy Group (AMLSG), 4-year relapse-free survival was 24.5% in t-AML and 39.5% in *de novo* AML and 4-year overall survival was 25.5% and 37.9% (age-stratified log-rank test *P* = .001 for both). In multivariate analysis, t-AML remained an adverse prognostic factor for death in complete remission, but not relapse, and overall survival in younger intensively treated patients. On the other hand it predicted relapse, but not death in complete remission, in older, less intensively treated patients. In more intensively-treated younger adults, treatment-related toxicity had a major negative impact on outcome, possibly reflecting cumulative toxicity of cancer treatment.^4^

An analysis matched for age, ECOG PS and additional cytogenetic abnormalities, compared *de novo* and therapy related Core Binding Factor (CFB) AML, carrying the recurrent chromosomal aberrations inv16 or t(8;21). An inferior treatment outcome was observed in patients with t-AML, with a median overall survival of 100 weeks compared to 376 weeks in *de novo* CBF AML (*P* = .001).^46^ The negative prognostic role of t-AML in inv16 AML has been confirmed also by the AMLSG prospective trials.^4^ On the other hand, favorable karyotype t-AML, including t(15;17) and t(8;21), treated according to standard protocols, had an outcome similar to *de novo* cases, indicating the dominant prognostic role of good karyotypes.^4^

## Treatment Implications

Besides biological factors associated to a more aggressive disease behavior, host related factors, including performance and functional status, cumulative toxicities and comorbity significantly impact on prognosis, treatment tolerability and complications. Both elderly and therapy-related AML are associated with reduced bone marrow hematopoietic cell reserve, due to physiological aging-related bone marrow impairment or to the effect of previous cytotoxic treatment, respectively. This hampers patients to overcome intensive treatment or makes recovery time longer than that of younger patients with *de novo* AML, so that most patients have to interrupt treatment after induction chemotherapy due to complications. Notwithstanding, t-AML patients with good performance status, favorable karyotypes and without significant comorbidities should still take advantage from standard therapy, since response rates are similar to younger patients with *de novo* AML. All these aspects should accurately be taken into account during treatment decision-making.

Before starting treatment, elderly leukemic patients should undergo comprehensive geriatric assessment (CGA) to identify problems that may interfere with cancer treatment, as recommended by the NCCN guidelines.^13^ Comorbidity burden significantly impacts on response rate, treatment-related toxicity and survival. The use of standardized comorbidity assessment tools can help to stratify patients and identify those who are less likely to benefit from standard therapies. Both the Charlson Comorbidity Index (CCI) and the Hematopoietic Cell Transplantation Comorbidity Index (HCT-CI) were able to identify patients with higher score associated with an adverse prognosis.^48^,^49^

Evaluation of physical function provides information independent of comorbidity in older patients with AML. Score systems, such as the Eastern Cooperative Oncology Group (ECOG) and the Karnofsky performance status scales and the Basic Activities of Daily Living (ADL) and Instrumental Activities of Daily Living (IADL) scales, can detect subclinical disability that might predict tolerability and response.^13^

Although the optimal therapy for older patients with AML remains debated, there is some evidence to guide decision making for individual patients. Older adults with newly diagnosed AML who present with any of the following characteristics are more likely to experience toxicity and less likely to benefit from standard induction chemotherapy: an ECOG score >2, significant comorbidity (CCI score >1, HCT-CI score >2), and poor risk cytogenetics. It would be reasonable to offer these patients supportive care alone, low-intensity therapy, or a clinical trial investigating novel agents. Alternatively, older adults with good functional status (ECOG score < 2, no impairment in IADLs), minimal comorbidity, and good-risk cytogenetics are likely to benefit from standard curative therapies regardless of chronologic age. The optimal treatment for the large majority of older adults falling between these two extremes is unclear.^5^,^13^

Similarly to elderly AML, patients with t-AML should be accurately evaluated for performace status, comorbidities, status of the primary disease and presence of complications from primary therapy, as well as for cytogenetic abnormalities. Patients with therapy-related acute promyelocytic leukemia (APL) should be treated as patients with *de novo* APL. Patients with t-AML associated with other favorable karyotypes should receive standard induction chemotherapy followed by high-dose cytarabine consolidation. Patients with indermediate and unfavorable cytogenetics who have an HLA-matched donor should be considered for allogeneic HCT. Supportive care or low dose chemotherapy remain the gold standard for patients with poor performance status.^50^,^51^

New investigational treatment approaches are under consideration for t-AML and elderly AML patients, including hypomethylating agents which induce significant response rates with limited toxicity in patients with unfavorable biologic factors and not susceptible of intensive chemotherapy.^5^,^13^,^50^,^51^

## Conclusions

The different biology of elderly AML favors the hypothesis of the involvement of specific pathogenetic pathways in this disease, similar to therapy-related AML. *De novo* AML in younger patients, especially those with “balanced” karyotypes, are thought to result from a limited number of mutational events restricting the diversity of leukemia subclones and leaving many cell functions relatively intact. In contrast, AML in the elderly may more often be the result of a string of mutational events leading to multiple leukemic subclones with the opportunity to develop multiple mechanisms of chemoresistance.^52^

Both elderly and t-AML take their origin by the physiological or treatment-induced aging process of bone marrow hematopoietic system and microenvironment, associated to reduced efficiency of DNA repair system, reduced hematopoietic reserve and immune surveillance, and prolonged exposure to carcinogens (environmental or cytotoxic agents and irradiation, respectively).

New therapeutic development are needed, because actual therapy strategies are limited due both to host-related factors responsible of poor tolerance to standard chemotherapy, and adverse biological features associated to treatment resistance.

## Figures and Tables

**Table 1:** Similarities between elderly and therapy-related AML

Similarities	AML in older patients *	t-AML**
Morphology and Clinics	Frequent history of a myelodysplastic phase and common myelodysplasia-related changes Lower WBC count	Frequent history of a myelodysplastic phase and common myelodysplasia-related changes (if previous exposure to alkylating agents and radiation) Lower WBC count
Cytogenetic	Frequent adverse cytogenetics (-5/5q-, -7/7q-, complex, monosomal)	Frequent adverse cytogenetics (-5/5q-, -7/7q-, complex, monosomal)
Molecular	Lower frequency of FLT3, NPM1, CEBPA mutation; Higher frequency of TET2, DNMT3A, IDH1 and IDH2 mutation No differences in RUNX1 and NRAS mutation frequency.	Lower frequency of FLT3, NPM1, CEBPA, TET2 mutation; Unknown DNMT3A, IDH1 and IDH2 mutation frequency. Higher RUNX1 mutation frequency No difference in NRAS mutation frequency
Biology	High MDR1 expression Distinct GEP Shorter telomere length compared to normal BM, but not to AML in younger patients	High MDR1 expression Distinct GEP Shorter telomere length Higher frequency of gene promoter hypermethylation

* Compared to AML in younger patients

** Compared to *de novo* AML
